# Structural, Mechanistic, and Functional Insights into an *Arthrobacter nicotinovorans* Molybdenum Hydroxylase Involved in Nicotine Degradation

**DOI:** 10.3390/molecules26144387

**Published:** 2021-07-20

**Authors:** Lei Wang, Xia Mu, Wenjin Li, Qin Xu, Ping Xu, Liyun Zhang, Yuebin Zhang, Geng Wu

**Affiliations:** 1 State Key Laboratory of Microbial Metabolism, The Joint International Research Laboratory of Metabolic & Developmental Sciences, School of Life Science and Biotechnology, Shanghai Jiao Tong University, Shanghai 200240, China; wanglei0806@sjtu.edu.cn (L.W.); xuqin523@sjtu.edu.cn (Q.X.); pingxu@sjtu.edu.cn (P.X.); 2 State Key Laboratory of Molecular Reaction Dynamics, Dalian Institute of Chemical Physics, Chinese Academy of Sciences, Dalian 116023, China; muxia@dicp.ac.cn; 3 State Key Laboratory of Medicinal Chemical Biology, College of Life Sciences, Nankai University, Tianjin 300350, China; liwenjin960531@163.com (W.L.); 019157@nankai.edu.cn (L.Z.)

**Keywords:** nicotine degradation, ketone dehydrogenase, Kdh, molybdenum hydroxylase, crystal structure, reaction mechanism, *Arthrobacter nicotinovorans*

## Abstract

*Arthrobacter nicotinovorans* decomposes nicotine through the pyridine pathway. 6-hydroxypseudooxynicotine 2-oxidoreductase (also named ketone dehydrogenase, Kdh) is an important enzyme in nicotine degradation pathway of *A. nicotinovorans*, and is responsible for the second hydroxylation of nicotine. Kdh belongs to the molybdenum hydroxylase family, and catalyzes the oxidation of 6-hydroxy-pseudooxynicotine (6-HPON) to 2,6-dihydroxy-pseudooxynicotine (2,6-DHPON). We determined the crystal structure of the Kdh holoenzyme from *A. nicotinovorans*, with its three subunits KdhL, KdhM, and KdhS, and their associated cofactors molybdopterin cytosine dinucleotide (MCD), two iron-sulfur clusters (Fe_2_S_2_), and flavin adenine dinucleotide (FAD), respectively. In addition, we obtained a structural model of the substrate 6-HPON-bound Kdh through molecular docking, and performed molecular dynamics (MD) and quantum mechanics/molecular mechanics (QM/MM) calculations to unveil the catalytic mechanism of Kdh. The residues Glu345, Try551, and Glu748 of KdhL were found to participate in substrate binding, and Phe269 and Arg383 of KdhL were found to contribute to stabilize the MCD conformation. Furthermore, site-directed mutagenesis and enzymatic activity assays were performed to support our structural and computational results, which also revealed a trend of increasing catalytic efficiency with the increase in the buffer pH. Lastly, our electrochemical results demonstrated electron transfer among the various cofactors of Kdh. Therefore, our work provides a comprehensive structural, mechanistic, and functional study on the molybdenum hydroxylase Kdh in the nicotine degradation pathway of *A. nicotinovorans.*

## 1. Introduction

China is a major tobacco production and processing country and has the largest number of smokers in the world. Nitrogen-containing heterocyclic aromatic compounds are pollutants for the environment and toxins for most living organisms. These pollutants are usually produced in the process of pesticide production, tobacco processing, coking industry, etc. [1]. Nicotine, a nitrogen-containing heterocycle, is contained in a large amount in tobacco-processing waste and is highly toxic. Nicotine may accumulate in human adipose tissue, cause long-term injury, and lead to cancer, teratoma, and other diseases [2]. Due to the electron-withdrawing effect of the nitrogen atom on the pyridine ring of nicotine, the electron density on carbon atoms of the pyridine ring decreases and makes it become a “π deficient” heterocycle, which is relatively stable and difficult to be effectively degraded through traditional physical and chemical approaches. Yet, some bacteria, such as *Arthrobacter* and *Pseudomonas*, possess enzymes which can effectively degrade pyridine-containing pollutants such as nicotine without harsh reaction conditions, providing a promising way to remove heterocyclic pollutants from the environment [3]. In the nicotine degradation pathways of *Arthrobacter* and *Pseudomonas*, a hydroxylase/monooxygenase first activates the aromatic ring by introducing a hydroxyl group onto it. Next, a dioxygenase breaks up the aromatic ring to produce an aldehyde or a carboxylic acid. Finally, amide hydrolases and isomerases complete the catabolism process [4]. Hydroxylases/monooxygenases usually achieve the oxidation of their substrates with the help of metal-containing cofactors such as heme, molybdopterin, iron-sulfur clusters, etc. [5].

**Scheme 1:**
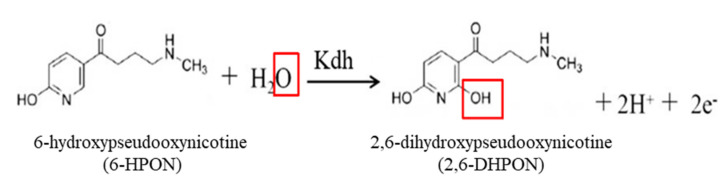
The reaction is Kdh-catalyzed.

Since the 1950s, more than 30 different bacteria have been found to degrade nicotine, and their nicotine-degradation mechanisms have been unveiled accordingly. For example, *Pseudomonas geniculata* N1 adopts a hybrid of pyridine and pyrrolidine pathways [6,7], *Pseudomonas putida* S16 uses the pyrrolidine pathway [8], and *Arthrobacter* sp. employs the pyridine pathway [9]. *Arthrobacter nicotinovorans* can use nicotine as the sole carbon and nitrogen source and employs the pyridine pathway for nicotine degradation [10]. In the nicotine degradation pathway of *A. nicotinovorans*, there are six enzymes involved: nicotine dehydrogenase (Ndh), 6-hydroxynicotine oxidase (6-HLNO and 6-HDNO), 6-hydroxypseudooxynicotine 2-oxidoreductase (or ketone dehydrogenase, Kdh), 2,6-dihydroxyl pseudooxide nicotine hydratase (DHPONH), 2,6-dihydroxypyridine hydroxylase (DHPH), and γ-N-methylamine butyrate oxidase (MABO) [9]. In the first step, Ndh catalyzes hydroxylation of the pyridine ring of nicotine to form 6-hydroxyl nicotine. Next, Kdh catalyzes a second hydroxylation of the pyridine ring of 6-HPON to produce 2,6-DHPON (Scheme 1) [11].

Both Kdh and Ndh belong to the family of molybdenum hydroxylases, which are characterized by their associated molybdopterin cofactors at the active site [12]. The molybdenum-containing molybdopterin cofactor is widely present in various organisms and is often found at the active sites of many enzymes that catalyze oxygen transfer. It was found that the oxygen atom in the product comes from water, not molecular oxygen [13,14]. Crystal structures of several molybdenum hydroxylases have been determined, indicating that molybdenum coordination spheres usually consist of 2 oxo-ligands, 2 ene-dithiolate sulfur atoms, and 1 catalytically critical sulfido-ligand [13,15,16,17]. Kdh is a heterotrimer containing three subunits. The 87 kDa KdhL subunit contains molybdopterin cytosine dinucleotide (MCD) cofactor, the 32 kDa KdhM subunit harbors a flavin adenine dinucleotide (FAD), and the 17.6 kDa KdhS subunit carries 2 Fe_2_S_2_ iron-sulfur clusters [18]. The structure of molybdo-iron-sulfur flavoproteins such as human aldehyde oxidoreductase (PDB code 4uhw), bovine milk xanthine dehydrogenase (PDB code1v97), carbon monoxide dehydrogenase (PDB code 1zxi), and quinoline 2-oxidoreductase (PDB code 1t3q) have been researched, and it was found that the distance between cofactors MCD, Fe_2_S_2_ and FAD was less than 15 Å [12,15,19,20]. In this work, we determined the crystal structure of the Kdh holoenzyme, including all its three subunits and their associated cofactors. By combining structural analysis, molecular docking, and enzyme activity assays, we identified residues of Kdh that play important roles in substrate binding. In order to understand how Kdh catalyzes the oxidation of 6-HPON to form 2,6-DHPON, a molecular dynamics simulation was performed to explore the stable binding mode of 6-HPON at the MCD site of Kdh, and a QM/MM simulation combined with the Nudged Elastic Band (NEB) method was used to investigate the potential of mean force (PME) along the NEB-optimized minimum energy path (MEP) of the intermediate states along the proposed reaction pathway. Therefore, this work provides a thorough structural, mechanistic, and functional study on the molybdenum hydroxylase Kdh involved in the degradation of the environmental pollutant nicotine by *A. nicotinovorans*.

## 2. Results and Discussion

### 2.1. Overall Structure of the Kdh Holoenzyme

In order to understand the molecular mechanism of Kdh, we coexpressed the three subunits of Kdh (KdhL, KdhM, and KdhS) in *Arthrobacter nicotinovorans* using the pAO1 plasmid [10]. The Kdh holoenzyme was purified through affinity chromatography and size-exclusion chromatography (Supplementary Figure S1) and crystallized in the space group *P*2_1_2_1_2_1_, with two molecules of the Kdh holoenzyme (a dimer of heterotrimers) per asymmetric unit. Through the molecular replacement approach, we determined the crystal structure of the Kdh holoenzyme to 3.4 Å resolution (Table 1), by using the structures of quinoline 2-oxidoreductase (Qor, PDB code 1T3Q) and bovine XDH (PDB code 1V97) as the searching model. The sequence of Kdh is homologous to Qor and XDH, with a sequence identity about 40% to Qor and 30% to XDH. Their tertiary structures are similar, with all showing a butterfly-shaped dimer [15] (Supplementary Figure S2). The final refined Rwork and Rfree factors were 28.7% and 32.6%, respectively. The electron density in the critical region is clear enough to support our following research (Supplementary Figure S3).

**Figure 1:**
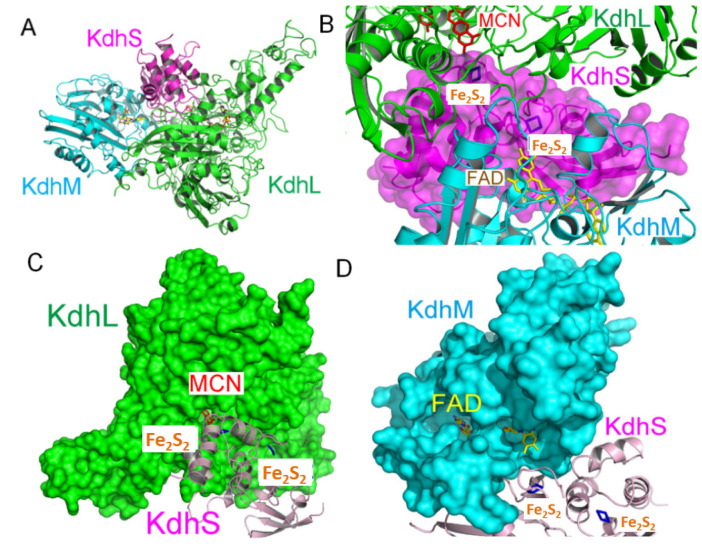
Crystal structure of Kdh. (**A**) Structure of the Kdh holoenzyme. KdhL, KdhM, and KdhS are colored in green, cyan, and magenta, respectively. Their associated cofactors are shown as stick representations. (**B**) The 2 Fe_2_S_2_ iron-sulfur clusters associated with KdhS are deeply buried and well-protected by the Kdh enzyme. The surface of KdhS is set as transparent. (**C**) The MCD cofactor associated with KdhL is also restricted from exterior access. The surface of KdhL is set as opaque. (**D**) The FAD cofactor associated with KdhM is relatively more open to the outside of the enzyme, allowing it to unload the electrons transferred from the substrate through the cofactor chain. The surface of KdhM is set as opaque.

In the structure, the overall size of the Kdh holoenzyme is 141.6 × 101.7 × 72.3 Å^3^. The smallest Kdh subunit, KdhS, is located in the middle, with the two larger subunits KdhL and KdhM poised at the two flanks. The KdhS subunit harbors two Fe_2_S_2_ iron-sulfur clusters (Figure 1A,B). Interestingly, these two iron-sulfur clusters are completely buried inside the amino acids; this feature is widespread in molybdo-iron-sulfur flavoproteins (Supplementary Figure S4), with KdhL and KdhS blocking their access to the exterior. This provides a protection for the two iron-sulfur clusters of KdhS against oxidation by oxygen in the air. Similarly, the molybdenum-containing MCD cofactor is also well protected by the amino acid residues of KdhL (Figure 1C). In contrast, the KdhM-bound FAD cofactor is relatively less restricted from the exterior (Figure 1D), which might be related the fact that FAD resides in the last link of the electron transfer chain and needs access to the outside of enzyme to unload the electrons deprived from the substrate.

### 2.2. Structure of Each Subunit of Kdh

**Figure 2:**
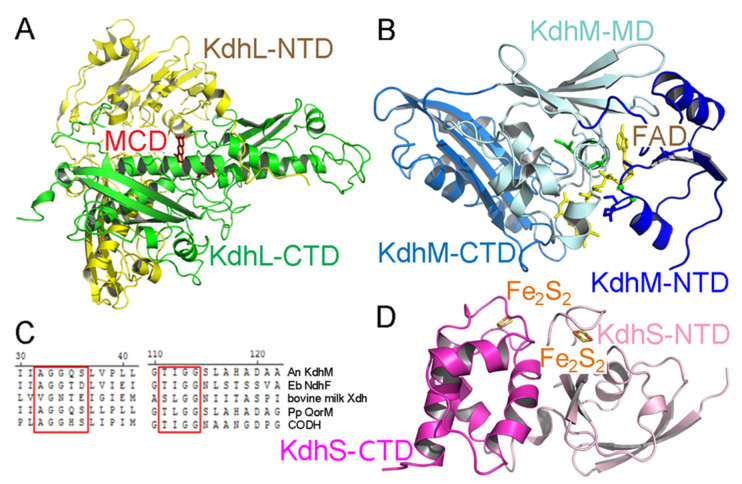
Structure of each subunit of Kdh. (**A**) Structure of KdhL. The N-terminal domain (NTD) and the C-terminal domain (CTD) of KdhL are colored in yellow and green, respectively. The MCD cofactor is colored in red. (**B**) Structure of KdhM. The NTD, middle domain (MD), and CTD of KdhM are colored in blue, pale cyan, and marine, respectively. The FAD cofactor is colored in yellow. Amino acid residues from the 2 FAD-binding motifs of KdhM are displayed in stick representation and colored in green. (**C**) Sequence alignment of the two conserved FAD-binding motifs from various molybdenum hydroxylases. Abbreviations: An KdhM: *Arthrobacter nicotinovorans* KdhM; Pp QorM: *Pseudomonas putida* 86 quinoline 2-oxidoreductase M subunit; Eb NdhF: *Eubacterium barkeri* NdhF; CODH: *Oligotropha carboxidovorans* carbon monoxide dehydrogenase. (**D**) Structure of KdhS. The NTD and CTD domains of KdhS are colored in pale pink and magenta, respectively. The two Fe_2_S_2_ are shown in stick representation.

The large subunit KdhL includes two domains. The N-terminal domain of KdhL (residues 1–416) mainly consists of β sheets and embraces the C-terminal domain of KdhS (residues 77–160). The C-terminal domain of KdhL (residues 417–790) is almost perpendicular to the N-terminal domain, and the MCD cofactor is at the center of their intersection. The C-terminal domain can be further divided into two subdomains. The first subdomain (residues 417–624) contains a long central α helix (residues 552–582) and a three-stranded β sheet. The second subdomain (residues 625–790) is composed of three β sheets and three α helices (Figure 2A).

The middle subunit KdhM contains 287 residues and can be divided into three domains (Figure 2B). The N-terminal domain (residues 1–61) comprises a three-stranded parallel β sheet flanked by two α helices and possesses one of the FAD-binding motifs ^32^AGGQS^36^ which is called the glycine motif [21]. This motif is strictly conserved in quinoline 2-oxidoreductase (Qor) [15], *however*, *in Eubacterium barkeri* NDH and *Oligotropha carboxidovorans* CODH, which possess an AGG sequence [16,22]. However, bovine milk XDH has only G [13] (Figure 2C). The middle domain (residues 62–172) is composed of six short α helices and a five-stranded antiparallel β sheet. The second glycine motif [19] of KdhM, ^111^TIGG^114^, is situated in the sixth α helix. This motif is conserved in *Eubacterium barkeri* NDH [16], CODH [22], Qor [15], and bovine milk XDH [13] (Figure 2C). The C-terminal domain (residues 173–287) is comprised of three α helices and a three-stranded antiparallel β sheet, and is not directly contact with FAD. In FAD, the isoalloxazine ring is the reaction position, which can accept two electrons and two protons. The isoalloxazine ring is well-protected by the KdhM subunit, especially the residue Y193 [15].

The small subunit KdhS has only 160 residues and bridges KdhL with KdhM. It can be divided into two domains (Figure 2B), with each of them binding an Fe_2_S_2_ iron-sulfur cluster. The first domain (residues 1–85) is composed of antiparallel β sheets and three short helices, with an iron-sulfur cluster coordinated by residues from loops connecting secondary structure elements. The second domain (residues 86–160) consists of four helices, with the loops between the helices associated with a second iron-sulfur cluster. The two iron-sulfur clusters are well shielded by KdhS (Supplementary Figure S4) and are further protected by KdhL and KdhM at both sides of KdhS (Figure 2B), not accessible by solvent or oxygen.

Some crystal structures for enzymes that involved in the nicotine degradation have been reported [4,7]; however, there are no crystal structures for Kdh. This is perhaps because Kdh is difficult to express, especially in terms of obtaining the purity and concentration necessary for crystallization. In our study, we determined the structure of Kdh, and despite the low resolution, the electron density maps of cofactors and important amino acids are clear enough for research. A sequence alignment and structural comparison with homologous enzymes showed that the spatial arrangement of cofactors in these enzymes was similar, and the residues involved in cofactor binding were conserved.

### 2.3. The Cofactors of Kdh Are Arranged in a Linear Fashion

**Figure 3:**
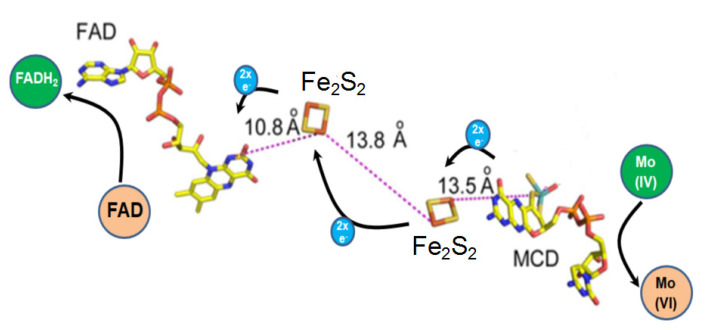
The cofactors of Kdh are arranged in a linear fashion, with distances between adjacent cofactors less than 14 Å. The MCD, the 2 Fe_2_S_2_ iron-sulfur clusters and the FAD cofactors of Kdh are shown in stick representations, with distances between adjacent cofactors labeled.

The three cofactors of Kdh are arranged in a linear fashion. MCD resides at one end of the line and FAD is located at the other end, with the two Fe_2_S_2_ iron-sulfur clusters staying in the middle. The distances between MCD and its nearby iron-sulfur cluster, that between the two Fe_2_S_2_ iron-sulfur clusters, and that between FAD and its neighboring iron-sulfur cluster are 13.5 Å, 13.8 Å, and 10.8 Å, respectively (Figure 3). Presumably, the Mo ion is oxidized from Mo (IV) to Mo (VI), resulting in two electrons being transferred to the two iron-sulfur clusters and then to FAD. The FAD, as the electron receptor, is reduced to FADH_2_. It has been found that in proteins involved in electron transfer, such as oxidoreductases, the distances between neighboring cofactors are usually less than 14 Å, which allows various cofactors to transfer electrons by the quantum tunneling effect without the need for direct contact [19,23].

### 2.4. The Substrate Channel of KdhL

**Figure 4:**
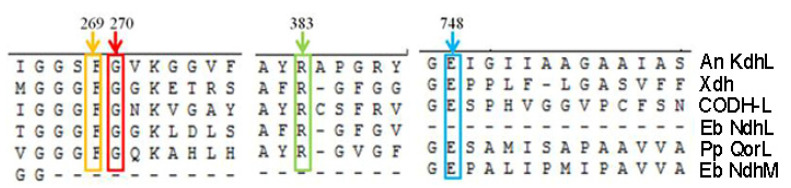
Conserved residues at the catalytic pockets of different molybdenum hydroxylases. Abbreviations—An KdhL: *Arthrobacter nicotinovorans* KdhL subunit; Xdh: bovine milk Xdh; Pp QorL: *Pseudomonas putida* 86 quinoline 2-oxidoreductase L subunit; Eb NdhL: *Eubacterium barkeri* NdhL subunit; CODH-L: *Oligotropha carboxidovorans* carbon monoxide dehydrogenase L subunit; Eb NdhM: *Eubacterium barkeri* NdhM subunit.

**Figure 5:**
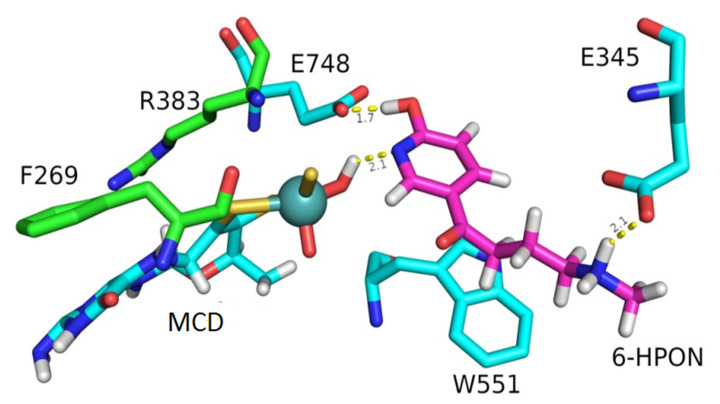
A structural model of the substrate 6-HPON bound at the active site of Kdh. E748, W551, and E345 of KdhL are directly involved in substrate binding. F269 and R383 of KdhL stabilize the conformation of MCD.

The overall structure and the spatial arrangement of MCD, FAD, and Fe_2_S_2_ are very similar to those of Qor and XDH. From the molecular docking study, we predict that 6-HPON preferentially binds to the MCD active site. There is a channel inside the KdhL, in which the MCD and 6-HPON lie (Supplementary Figure S5). The amino acids that may interact with MCD and 6-HPON are shown. The residues G270, F269, P385, R383, and S552 may stabilize MCD, while the residues F269, P385, and W551 may provide hydrophobic enviroment for 6-HPON binding. The residues G270, G273, G274, Q343, A344, and E345 are located near the entrance of the channel, and may facilitate substrate entry and product release (Supplementary Figure S6). The residues at the active site of KdhL directly interacting with the MCD cofactor are highly conserved in other molybdenum hydroxylases (Figure 4, Supplementary Figure S7). To understand how Kdh recognizes its substrate 6-HPON, we further obtained a model of 6-HPON bound Kdh through molecular docking (Figure 5). The active site loop, ^381^AYRAPG^386^, is ~4 Å away from the molybdenum atom of the MCD cofactor. This motif is conserved in the molybdenum hydroxylase family, for example in *Pseudomonas putida* 86 quinoline 2-oxidoreductase (^369^AYRGVG^374^) [15], *Eubacterium barkeri* Ndh (^349^AFRGFG^354^) [16], bovine milk Xdh (^910^AFRGFG^916^) [13], and *Eubacterium Oligotropha carboxidovorans* CODH (^385^AYRCSF^390^) [22], with slight changes in residues of this loop depending on specific substrates [20]. The residue G270, a small amino acid strictly conserved in other molybdenum hydroxylases, is only 3.9 Å away from the molybdenum atom. The phenyl ring of F269, another highly conserved residue, is coplanar with the pterin ring of MCD and forms a π-π stacking interaction. E748 has strictly conserved molybdo-iron-sulfur flavoproteins, and presumably functions as a general base abstracting the proton and initializing the reaction [13]. Furthermore, both W551 and E345 are adjacent to the substrate 6-HPON, and are not conserved. The channel of each enzyme adapts to its specific substrate. Residues such as G470, A259, L334, and T332 are located in the channel, so it is easy for the large substrate Qor to pass [15]. G273, E345, and L347 of Kdh correspond to A259, T332, and L334 of Qor, making the channel permeable. W551 of Kdh corresponds to Y545 of Qor.

To confirm our structural and modeling results, we performed site-directed mutagenesis on Kdh and measured the enzymatic activity of these Kdh mutants. Mutation of R383A decreased the *k*_cat_ value to 70% and increased the *K*_m_ value by 1.7-fold (Table 2). Mutation of F269A decreased the *k*_cat_ value to 25% and increased the *K*_m_ value by 2.1-fold (Table 2). In addition, the single point mutants of E748A, W551A, and E345A all completely lost the enzymatic activity (Table 2).

### 2.5. Catalytic Mechanism of Kdh

**Figure 6:**
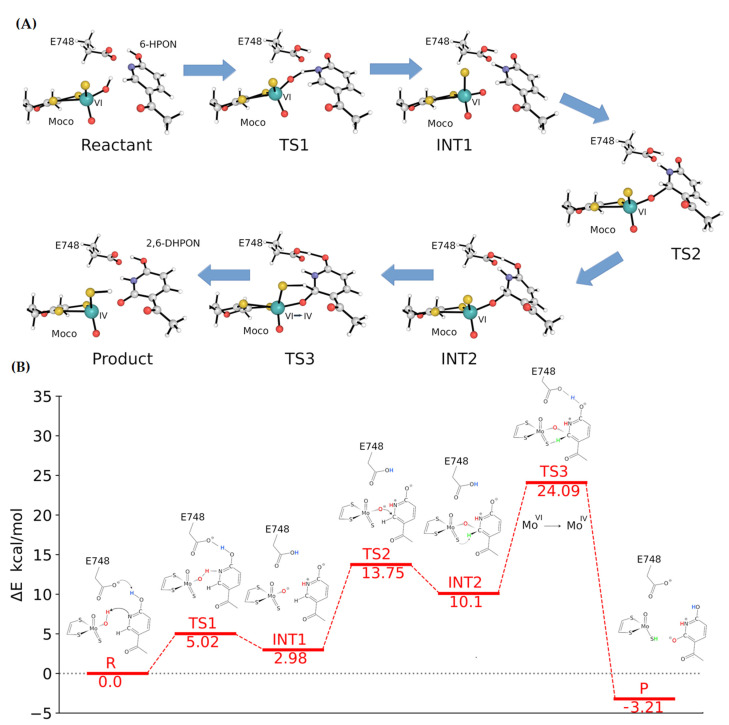
Reaction mechanism and free energy profile of Kdh-catalyzed reaction. (**A**) Our proposed reaction mechanism of Kdh-catalyzed oxidation 6-HPON, with KdhL-E748 playing an important role in the reaction. (**B**) Free energy profile of the oxidation of 6-HPON to 2,6-DHPON. This reaction consists of 3 sequential stages which are initialized by a proton transfer from Table 1. nitrogen atom of 6-HPON. The first process is also accompanied by a concerted proton transfer from the hydroxyl group of 6-HPON to the carboxylate group of E748, which maintains the charge balance of the substrate, supporting the hypothesis that E748 is involved in the catalytic process as a general base. The final step is the rate-limiting step of the whole catalytic process with an activation energy of 24.09 kcal/mol, corresponding to the hydride transfer from the pyridine C2 carbon atom in the tetrahedral geometry to the sulfur S1 of the MCD.

In order to understand the catalytic mechanism of Kdh, a molecular dynamics (MD) simulation was performed to explore the stable binding mode of 6-HPON at the MCD site of Kdh, and a QM/MM simulation combined with the Nudged Elastic Band (NEB) method [24] was used to investigate the potential of mean force (PME) along the NEB optimized minimum energy path (MEP) of the intermediate states along the proposed reaction pathway. The starting binding pose of 6-HPON was initially obtained from molecular docking and MD simulation and was used to equilibrate the binding state. After 500 ns of all-atom MD simulation of the Kdh heterotrimers, a snapshot of stable binding state was obtained from our MD simulation (Figure 6). The local environment of the 6-HPON binding site indicates that W551 plays a significant role in keeping the substrate steady. Meanwhile, E345 provides salt-bridge interaction with the tail amino group of 6-HPON. This binding mode is in agreement with previous reports for other molybdenum hydroxylases, such as bovine milk xanthine oxidoreductase in complex with its inhibitor FYX-051, and the sialic acid-bound xanthine dehydrogenase. 

Only 6-HPON with positively charged amino group could be stabilized during our MD simulation, whereas 6-HPON with a neutral amino group was released quickly from the binding site within 50 ns of our MD simulation. The conserved E748 maintains the stable binding of the substrate via a hydrogen-bonding interaction between E748 carboxylate and 6-HPON hydroxyl groups. We concluded that E748, W551, and E345 of KdhL directly take part in the binding of the substrate. Indeed, the KdhL mutants E748A and W551A lost their enzymatic activities completely (Table 2). E1261 of bovine XDH was regarded as a general base by abstracting the proton from Mo-OH, and then the protonated E1261 was stabilized by forming a hydrogen bond with the substrate [13]. In our study, we found that the proton of Mo-OH was transferred to the N1 of 6-HPON pyridine group, and then KdhL-E748 accepted the proton from 6-HPON hydroxyl group (Supplementary movie; Figure 6). Generally speaking, glutamate is regarded as the key active site residue for the activation of OH ligands to attack the substrates [25]. KdhL-E345 provides a salt-bridge interaction with the tail amino group of 6-HPON (Figure 5). KdhL-F269 and KdhL-R383 are not directly involved in substrate binding, and their main roles are to stabilize the conformation of MCD (Figure 5). The measured enzyme activity of various Kdh mutants confirmed our hypothesis (Table 2).

A further QM/MM simulation demonstrated that the reaction consists of three sequential stages which are initiated by a proton transfer from the hydroxyl group of MCD to the pyridine N1 nitrogen atom of 6-HPON. This first process is also accompanied by a concerted proton transfer from the hydroxyl group of 6-HPON to the carboxylate group of E748, which functions as a general base and maintains the charge balance of the substrate. The transition state 1 (TS1) exhibits an activation energy barrier of 5.02 kcal/mol, and the products of this step have a reaction energy of 2.98 kcal/mol. Protonation of the N1 nitrogen atom also increases the carbocation character of the pyridine C2 carbon of 6-HPON, thus facilitating the next reaction process: the nucleophilic attack of hydroxyl oxygen O1 of the MCD towards the pyridine C2 carbon atom. During the nucleophilic attack, a tetrahedral intermediate state (INT2) was identified in which either the bond between the hydroxyl oxygen O1 of the MCD and the C2 carbon atom or the bond between the hydroxyl oxygen O1 of the MCD and the Mo ion are fully formed or broken, with the bond lengths of 1.48 Å for the O1–C2_6__-HPON_ bond and 1.90 Å for the O1–Mo bond, respectively. A tetrahedral angle of 103.7° for O1–C2_6__-HPON_–HC2 was observed in the transition state 2 (TS2) geometry, and the activation energy barrier was 13.75 kcal/mol. The final step of the reaction is the hydride transfer from the pyridine C2 carbon atom in the tetrahedral geometry to the sulfur S1 of the MCD. This step is the rate-limiting step of the whole catalytic process, with an activation energy of 24.09 kcal/mol. The localized orbital analysis [26] indicated that the oxidation state of Mo ion at transition state 3 (TS3) experienced a transition from Mo^VI^ to Mo^IV^, which is consistent with the findings that the hydride transfer is the rate-limiting step in aldehyde oxidase (AOX) [19]. In the final product 2,6-DHPON, formation of the bond between the hydroxyl oxygen O1 of MCD and the pyridine C2 carbon is completed, and the pyridine ring retains the planar geometry after the hydrogen transfers to the sulfur S1 of the MCD. Kdh-E748 releases the proton on the carboxylate group and gives it back to the hydroxyl group of 2,6-DHPON at the 6 position. The final product has a reaction energy of −3.21 kcal/mol, indicating that the whole reaction is exergonic (Figure 6).

### 2.6. Effect of pH on the Enzymatic Activity of Kdh

In order to determine the optimal pH for the Kdh-catalyzed reaction, we examined the catalytic efficiency of Kdh in the PBS buffer or the Tris-HCl buffer at different pH, and found that there was a trend of increasing catalytic efficiency with the increase in the buffer pH. When the PBS buffer was used, the *k*_cat_/*K*_M_ value was 0.08 ± 0.04 s^−1^μM^−1^, the *K*_M_ value was 64.67 ± 11.57 μM, and the *k*_cat_ value was 5.13 ± 0.41 s^−1^ at pH 6.4, while the *k*_cat_/*K*_M_ value increased to 0.45 ± 0.13 s^−1^μM^−1^ and the *k*_cat_ value increased to 10.58 ± 0.22 s^−1^, while the *K*_M_ value decreased to 23.41 ± 1.76 μM at pH 7.4. Furthermore, the *k*_cat_/*K*_M_ value was raised to 0.72 ± 0.13 s^−1^μM^−1^, the *K*_M_ value decreased to 12.5 ± 0.91 μM, and the *k*_cat_ value decreased to 9.03 ± 0.12 s^−1^ at pH 8.0 (Table 3). Similarly, when the Tris-HCl buffer was used, the *k*_cat_/*K*_M_ value was enhanced from 0.24 ± 0.07 to 0.42 ± 0.10 and then to 0.60 ± 0.12 s^−1^μM^−1^, and the *k*_cat_ value increased from 9.09 ± 0.15 to 9.28 ± 0.08 and then to 9.47 ± 0.13 s^−1^ when the pH changed from 6.8 to 7.4 and then to 8.0. The *K*_M_ value decreased from 37.71 ± 2.14 μM to 15.09 ± 1.44 μM when pH changed from 6.8 to 8.5. However, when the Tris-HCl at pH 8.5 was used, the *k*_cat_/*K*_M_ and *k*_cat_ values decreased slightly (Table 3, Supplementary Figure S8). Therefore, we concluded that pH 8.0 is the optimal buffer pH for Kdh, which is also the buffer pH in the crystallization condition of Kdh. The optimal pH measurements of enzymes help to guide the functional research.

**Figure 7:**
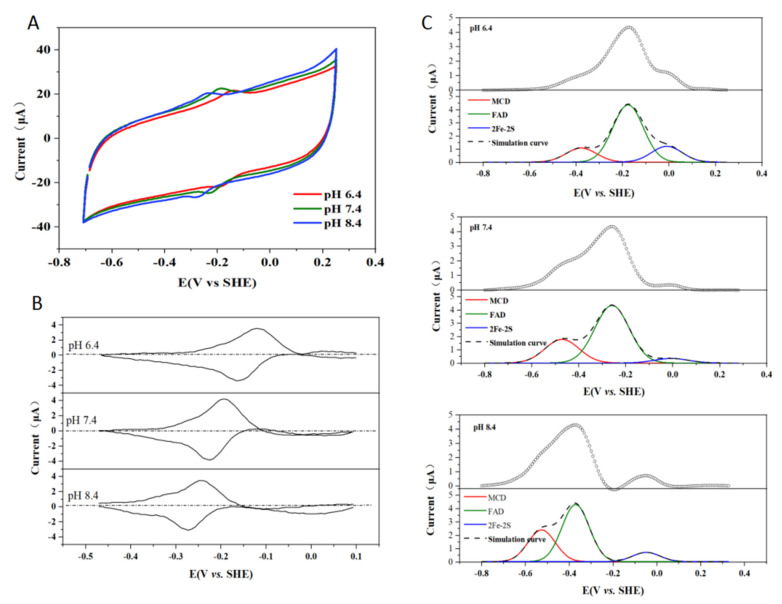
Cyclic voltammogram of Kdh. (**A**) Cyclic voltammogram of Kdh (0.1 mg/mL) in 10 mM Tris buffer with 0.9% NaCl at various pH. The voltammetric rate was 0.5 V·s^−1^ and the temperature was 25 °C. (**B**) Voltammograms of Kdh at a PGE electrode (with an area of 0.04 cm^2^) in the absence of 6-HPON, corrected for non-faradaic background current. The scan rate was 0.5 V·s^−1^ (analogue mode), and the temperature was 25 °C. The electrolyte consisted of 0.1 mg/mL bulk Kdh in 10 mM Tris buffer containing 0.9% NaCl. (**C**) Deconvolution of the oxidation current, corrected for non-faradaic background current, at pH 6.4, pH 7.4, and pH 8.4. Other conditions are as in (**B**).

In order to study the electron transfer among MCD, iron-sulfur clusters, and FAD, we performed electrochemical experiments of Kdh in the Tris-HCl buffer of pH 6.4, pH 7.4, and pH 8.4 [27]. When only the Kdh enzyme and no substrate 6-HPON was present, the electrical signal recorded by the electrochemical workstation indicated the electron transfer characteristic of the enzyme itself (Figure 7A). When the background signal of the graphite electrode was deducted, the result showed that the electron transfer of the enzyme itself was affected by the pH, as could be seen from the peak position shifting to the left with increasing pH (Figure 7B). All potentials were converted to standard hydrogen potentials at 25 °C. Using the Origin software to deconvolute, we could identify cofactor peaks corresponding to the potential of the cofactors with the corresponding pH (Figure 7C). At pH 8.4, the peak potential of MCD was −0.529 eV, that of FAD was −0.375 eV, and that of Fe_2_S_2_ was −0.045 eV. At pH 7.4, the peak potential of MCD was −0.466 eV, that of FAD was −0.260 eV, and that of Fe_2_S_2_ was −0.01 eV. At pH 6.4, the peak potential of MCD was −0.382 eV, that of FAD was −0.176 eV, and that of Fe_2_S_2_ was −0.015 eV. The results of redox potentials suggested that MCD, Fe_2_S_2_, and FAD involved in electron transfer. It has been accepted that oxidoreductases with multiple cofactors transfer electrons through the quantum tunneling effect [23]. However, as theoretical formulations regarding electron transfer between cofactors are scarce, next we will deeply study this with the help of bioinformatics technology. Meanwhile, we will continue to improve the resolution and obtain crystal complex of Kdh and 6-HPON.

## 3. Materials and Methods

### 3.1. Cloning and Purification

The *kdhL* and *kdhMS* genes were kindly provided by Dr. Roderich Brandsch from University of Freiburg. Sal I and Xba I were used to cleave the pART2 vector [28] and the *kdhMS* fragment was amplified by PCR. The target gene fragment was then ligated with the linearized pART2 vector by T4 DNA ligase at 16 °C overnight. The recombinant plasmid was transformed into *E*. *coli* DH5α and incubated at 30 °C in the LB/agar medium containing kanamycin. Single colonies were validated by PCR screening and sequencing. In the same way, the *kdhL* gene was cloned into the plasmid pART2-*kdhMS* to obtain the pART2-*kdhLMS* plasmid.

Thus, 100 ng of pART2-*kdhLMS* plasmid and 50 μL of competent cells of *A. nicotinovorans* pAO1 [29] were mixed and incubated on ice for 5–10 min, and then transferred to the precooled electric transfer cup under the following conditions: electric field of 1.5 kV/mm, resistance of 400 Ω, and electrical capacity of 25 μF. The transformed cells were inoculated to 800 μL LB (with 0.5 mol sorbitol) and cultured at 30 °C, 120 rpm/min, for 8 h. Then, 100 μL of the cells were plated onto LB/agar medium containing 140 mg/L kanamycin, and cultured in a 30 °C incubator overnight. Positive transformants were selected and cultured in the citric acid medium containing kanamycin (950 mL citric acid media + 50 mL trace elements). Nicotine (1 mg/L) was added to the cells, which were further cultured at 30 °C, 180 rpm for 30 h. Bacteria were then harvested and centrifuged at 4000 rpm for 20 min. The pellet was resuspended in 25 mM Tris-HCl (pH 8.0), 20 mM imidazole, and 300 mM NaCl, supplemented with β-mercaptoethanol (1:1000) and 200 μM PMSF. The bacteria were lysed by ultrasonication, and the lysate was loaded onto a Ni^2+^-NTA column, which was then washed with a buffer of 25 mM Tris-HCl, pH 8.0, 20 mM imidazole, and 300 mM NaCl. The target protein Kdh was eluted by serial gradient of imidozole, concentrated, and further purified by the Superdex 200 HiLoad 16/60 column (GE Healthcare), which was pre-equilibrated with 15 mM Tris-HCl, 150 mM NaCl, 2 mM TCEP, and 2 mM DTT. The main peak fractions were concentrated to 10 mg/mL, frozen in liquid nitrogen, and stored at −80 °C.

### 3.2. Data Collection and Structure Determination

Crystals of the Kdh were grown at 14 °C for one week by the hanging-drop vapor-diffusion method. The reservoir solution contained 1% tryptone, 0.1 M Tris-HCl (pH 8.0), and 25% PEG 3350. Crystals were transferred to the crystallization buffer supplemented with 35% PEG 3350 before being flash-frozen. An X-ray diffraction dataset at 3.4 Å was collected at the beamline BL17U1 (SSRF, Shanghai, China), using an ADSC Quantum 315r CCD area detector(ADSC, CA, USA). The diffraction data were processed using the HKL3000 software [30]. The structure of Kdh was determined by the molecular replacement method with Phaser [31], using the structure of quinoline 2-oxidoreductase (PDB code 1T3Q) as a searching model. Data collection and refinement statistics are listed in Table 1. The structure was validated with PROCHECK, and the structure figures were produced by using PyMol [32].

### 3.3. Construction of Mutants

Point variants of F269A, R383A, W551A, E345A, and E748A of KdhL were constructed by the whole-plasmid PCR and DpnI digestion method. The plasmids were confirmed to be correct by DNA sequencing and then expressed. Proteins of the Kdh point variants were purified using the same procedure for the WT protein. The enzymatic activities of all variants were tested in the Tris-HCl, pH 8.0 buffer unless specified otherwise.

### 3.4. Enzyme Activity Assays

Proteins of WT and various point variants of Kdh were purified for the in vitro enzymatic activity assay. The substrate 6-HPON was purchased from the Sundia MediTech Company, Ltd. The enzymatic activity of Kdh was examined under aerobic conditions by a UV-visible spectrophotometer at OD_600_ using phenazine methosulfate (PMS) and 2,6-dichlorophenol indophenol (DCIP) as artificial electron acceptors to monitor 6-HPON oxidation. Assays were carried out in a quartz cuvette (1 cm optical path) using a UV-visible spectrophotometer 2550 (Shimadzu Corporation, Kyoto, Japan). Firstly, 0.1 mM 6-HPON and 0.1 mM Kdh were respectively scanned at 200–600 nm wavelengths. Kdh activity was detected by observing the reduction of DCIP at 600 nm (ε = 21 mM^−1^cm^−1^). Here, 1 unit of enzyme activity was defined as the reduction of 1 μmol DCIP per minute at 30 °C [33]. The total volume of the reaction was 800 µL. The buffer used was either 10 mM phosphate buffer (PBS, pH 6.4, pH 7.4, or pH 8.0) or 10 mM Tris-HCl buffer (pH 6.8, pH 7.4, pH 8.0, or pH 8.5). The concentrations of the substrate 6-HPON used were 6.25 μM, 15.62 μM, 31.25 μM, 46.875 μM, 62.5 μM, 78.125 μM, 93.75 μM, 125 μM, and 187.5 μM. The concentrations of PMS and DCIP used in the reaction were 500 μM and 50 μM, respectively. The reaction was started by the addition of 40 μL, 0.05 μg/μL Kdh enzyme. The absorbance was determined 20 s after the enzyme was added. Three parallel experiments were performed for each concentration.

### 3.5. Electrochemical Measurement

For electrochemical characterization, all measurements were recorded in an anaerobic glove box containing a N_2_ atmosphere (O_2_ < 2 ppm, Mikrouna). Measurements were carried out using an Autolab potentiostat (PGSTAT101) controlled by Nova software (EcoChemie) in a 3-electrode configuration, which consisted of an Ag/AgCl reference electrode in 0.1 M sodium chloride with ultrapure water (Milli-Q, 18 MΩ cm) and a Pt wire counter electrode. Cyclic voltammograms (CV) analysis was performed at a scan rate of 0.5 V·s^−1^. All potentials were reported with reference to the standard hydrogen electrode (SHE), based on a potential of 197 mV for Ag/AgCl reference electrode at 25 °C.

### 3.6. MD Simulation and QM/MM Calculation

The crystal structure of Kdh determined in the present work was used as an initial conformation to perform all-atom molecular dynamics (MD) simulation using Amber18 package. The AMBER ff14sb force field [34] was used. The parameters for the substrate 6-HPON (with a positively charged amino group or neutral amino group) and molybdopterin cofactor were prepared using the GAFF force field [35]. The Gaussian 09 package [36] with B3LYP/6-31G* method was employed to perform geometry optimization of compounds and the restrained electrostatic potential (RESP) approach was used to assign partial charges. The parameters for the cytosine dinucleotide and flavin adenine dinucleotide (FAD) were obtained from the R.E.DD.B force field libraries [37] (F-91). The parameters for the MCD cofactor and Fe_2_S_2_ clusters were prepared using MCPB.py [38], which is a python-based metal center parameter builder in Ambertools. The system was solvated using TIP3P water with a thickness of 12 Å and the Na^+^ ions were used as counter ions to neutralize the system, yielding a simulation system with a total of 330,588 atoms in a starting dimension of 154.19 Å × 157.00 Å × 154.68 Å. The system was initially refined using 500 steepest descent steps before switching to conjugate gradient energy minimization and gradually heated to 300 K within 2 ns. The positional restraints were exerted on the backbone of the protein with a weight of 10 kcal/mol·Å^2^ during the energy minimization and heating process. Then, the restraints were released gradually within 6 equilibration steps under constant pressure and temperature (NPT) ensemble. The hydrogen mass repartitioning was set to 4 amu to enable an integration step of 4 fs for the simulations. Five individual production runs were conducted using a 10 Å cutoff distance for the Lennard-Jones interaction, and the Particle Mesh Ewald (PME) summation method was used for calculating Coulomb interactions. As a comparison, MD simulation using the substrate 6-HPON with neutral tail amino group was also performed. However, we found this substrate would be released quickly from the binding site within 50 ns of our simulation. Therefore, these settings were terminated after 50 ns. Only the 6-HPON with a positively charged amino group would be stabilized in the binding site; hence, 5 × 500 ns MD simulations using the positively charged amino group of 6-HPON were conducted for further analysis and a snapshot of the stable binding mode was used for QM/MM simulation. In the QM/MM calculation, the MM region was truncated within 15 Å around the MCD, and the substrate 6-HPON, the MCD without its phosphate group, and the side chain of E748 were taken account into the QM region. The density functional theory (DFT) with the B3LYP hybrid functional [39] was used for QM atoms. The SDD effective core potential [40] was used in Mo and the def2-SVP basis sets [41] were used for the rest of the QM atoms. The Nudged Elastic Band method implemented in ORCA [24] was employed for investigating the potential of mean force (PME) along the minimum energy path (MEP) of the intermediate states along the proposed reaction pathway.

## 4. Conclusions

In this study, we determined the crystal structure of Kdh from *Arthrobacter nicotinovorans* and investigated the molecular mechanism of how Kdh recognizes its substrate 6-HPON and the oxidation mechanism of 6-HPON to 2,6-DHPON. Site-directed mutagenesis and enzymatic activity assays were performed to support our structural and computational results. Bioengineering methods using bacteria, crude extract, or purified enzymes in the nicotine degradation pathway can be used to treat tobacco waste and degrade nicotine. The enzyme method to treatment nicotine pollutants is helpful because enzymes can be used repeatedly and efficiently.

## Figures and Tables

**Table 1:** Data collection and refinement statistics.

Data Collection	Kdh
Beamline	BL17U1 (SSRF)
Space group	*P*2_1_2_1_2_1_
Unit cell parameter	
*a*, *b*, *c* (Å)	112.86, 126.79, 294.49
α, β, γ (°)	90, 90, 90
Resolution (Å)	50.0–3.4
R_merge_ (outer shell)	0.122 (0.693)
I/σ_I_ (outer shell)	11.3 (1.0)
CC_1/2_	0.512
Completeness (%) (outer shell)	100 (100)
Redundancy (outer shell)	12.2 (10.6)
Refinement	
Resolution (Å)	147.2–3.4
Number of reflections	50,560
R_work_/R_free_	28.7%/32.6%
Number of atoms	18676
B factors (Å^2^)	67.88
RMSD bond length (Å)	0.0097
RMSD bond angles (°)	1.7528
Ramanchandran plot	
Favored (%)	83.6
Allowed (%)	12.2
Disallowed (%)	4.1

R_merge_ = Σ_h_Σ_i_ |*I*_h,i_ − *I*_h_|/Σ_h_Σ_i_ *I*_h,i_ for the intensity (*I*) of observation i of reflection h. R factor = Σ||*F*_obs_| − |*F*_calc_||/Σ|*F*_obs_|, where *F*_obs_ and *F*_calc_ are the observed and calculated structure factors, respectively. R_free_ = R factor calculated using 5% of the reflection data chosen randomly and omitted from the start of refinement. RMSD = root mean square deviation. Data for the highest resolution shell are shown in parentheses.

**Table 2:** Enzymatic activity assay for wild-type (WT) and mutant Kdh enzymes. The assays were carried out in Tris-HCl, pH = 8.0.

WT and Mutants	*K*_M_ (μM)	*k*_cat_ (s^−1^)	*k*_cat_/*K*_M_ (s^−1^μM^−1^)
WT	12.5 ± 0.9	9.03 ± 0.12	0.72 ± 0.13
E748A	N.D.	N.D.	N.D.
F269A	25.98 ± 2.73	2.30 ± 0.07	0.09 ± 0.03
W551A	N.D.	N.D.	N.D.
R383A	21.4 ± 2.3	6.32 ± 0.16	0.30 ± 0.07
E345A	N.D.	N.D.	N.D.

N.D. indicates that the enzyme activity was not detected.

**Table 3:** The effect of pH on the enzymatic activity of Kdh.

Buffer	*K*_M_ (μM)	*k*_cat_ (s^−1^)	*k*_cat_/*K*_M_ (s^−1^μM^−1^)
PBS, pH 6.4	64.67 ± 11.57	5.13 ± 0.41	0.08 ± 0.04
PBS, pH 7.4	23.41 ± 1.76	10.58 ± 0.22	0.45 ± 0.13
PBS, pH 8.0	12.5 ± 0.91	9.03 ± 0.12	0.72± 0.13
Tris-HCl, pH 6.8	37.71 ± 2.14	9.09 ± 0.15	0.24 ± 0.07
Tris-HCl, pH 7.4	22.29 ± 0.81	9.28 ± 0.08	0.42 ± 0.10
Tris-HCl, pH 8.0	15.88 ± 1.08	9.47 ± 0.13	0.60 ± 0.12
Tris-HCl, pH 8.5	15.09 ± 1.44	8.92 ± 0.28	0.59 ± 0.19

## Data Availability

Data supporting the reported results will be available with the corresponding author(Geng Wu).

## Supplementary Materials

The following are available online. Figure S1: Purification of the Kdh holoenzyme. Figure S2: Overall structure of the Kdh holoenzyme and compare the structures of Kdh, XDH and Qor. Figure S3: Electron density around the Kdh cofactors and important amino acids near the MCD of the large subunit. Figure S4: The 2 Fe_2_S_2_ iron-sulfur clusters are completely wrapped inside KdhS. Figure S5: Both cofactor MCD and substrate 6-HPON are shown sitting in the channel. Figure S6: Putative the amino acids that interact with MCD and 6-HPON. Figure S7: Sequence alignment result of different molybdenum hydroxylases. Figure S8: The catalytic efficiency of Kdh in PBS/Tris-HCl buffer of different pH. Table S1: Strains, plasmids, and primers used in this study, Video S1: The reaction movie.

## Accession Code

The structure of the Kdh has been deposited in the Protein Data Bank with the PDB code 7DQX.
