# Supplementary material for: Structural, Mechanistic, and Functional Insights into an Arthrobacter nicotinovorans Molybdenum Hydroxylase Involved in Nicotine Degradation
Source: Molecules. 2021 Jul 20;26(14):4387. doi: 10.3390/molecules26144387 (PMC8305194; doi:10.3390/molecules26144387)
Supplement: Supplementary file 1 [file molecules-26-04387-s001.zip › molecules-1282361 -si/molecules-1282361 -si.pdf]

Supplementary Figures:

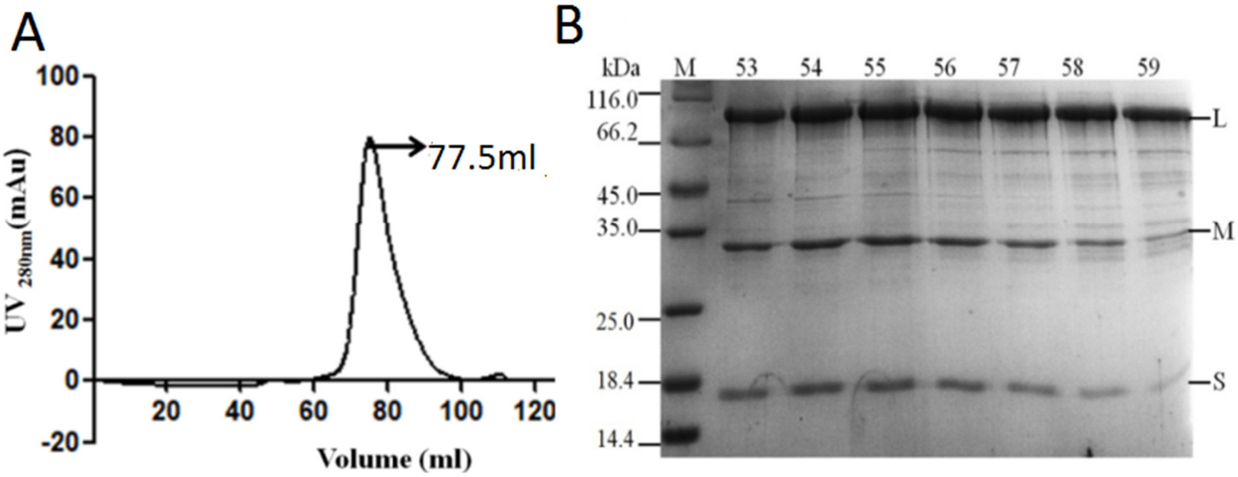

Supplementary Figure 1. Purification of the Kdh holoenzyme. (A) Kdh is monomeric in solution, with a molecular mass of about 140 kDa. Elution volume of Kdh is 77.5 mL through size-exclusion chromatography on superdex 200 HiLoad 16/60. (B) SDS-PAGE analysis of the fractions from size-exclusion chromatography. Fractions 53-59 are the elution tubes corresponding to the peak. L, M, and S are the large, middle and small subunits of Kdh, respectively.

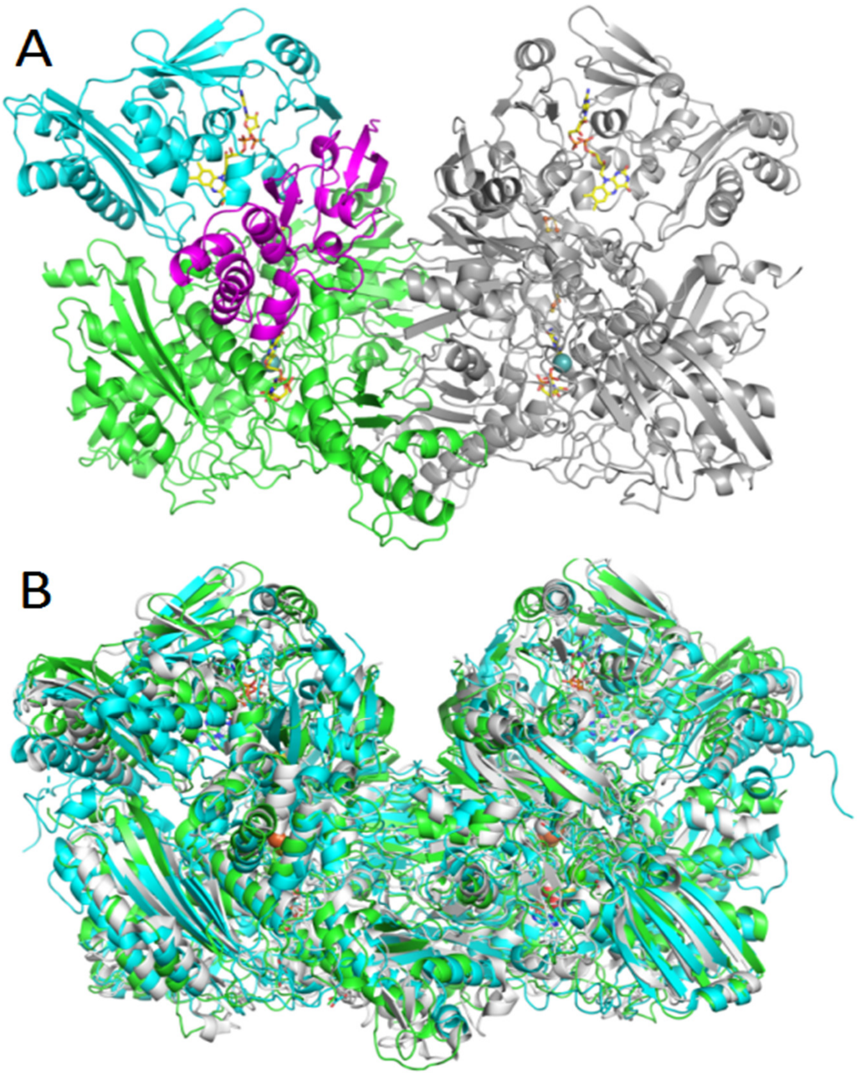

Supplementary Figure 2. (A) Overall structure of the Kdh holoenzyme (PDB code: 7DQX). (B) Compare the structures of Kdh, XDH and Qor. A stereoview of Kdh (green), Bovine XDH (PDB code: 1V97; cyan) and Qor (PDB code: 1T3Q; white), the superimposition was done on the whole molecule.

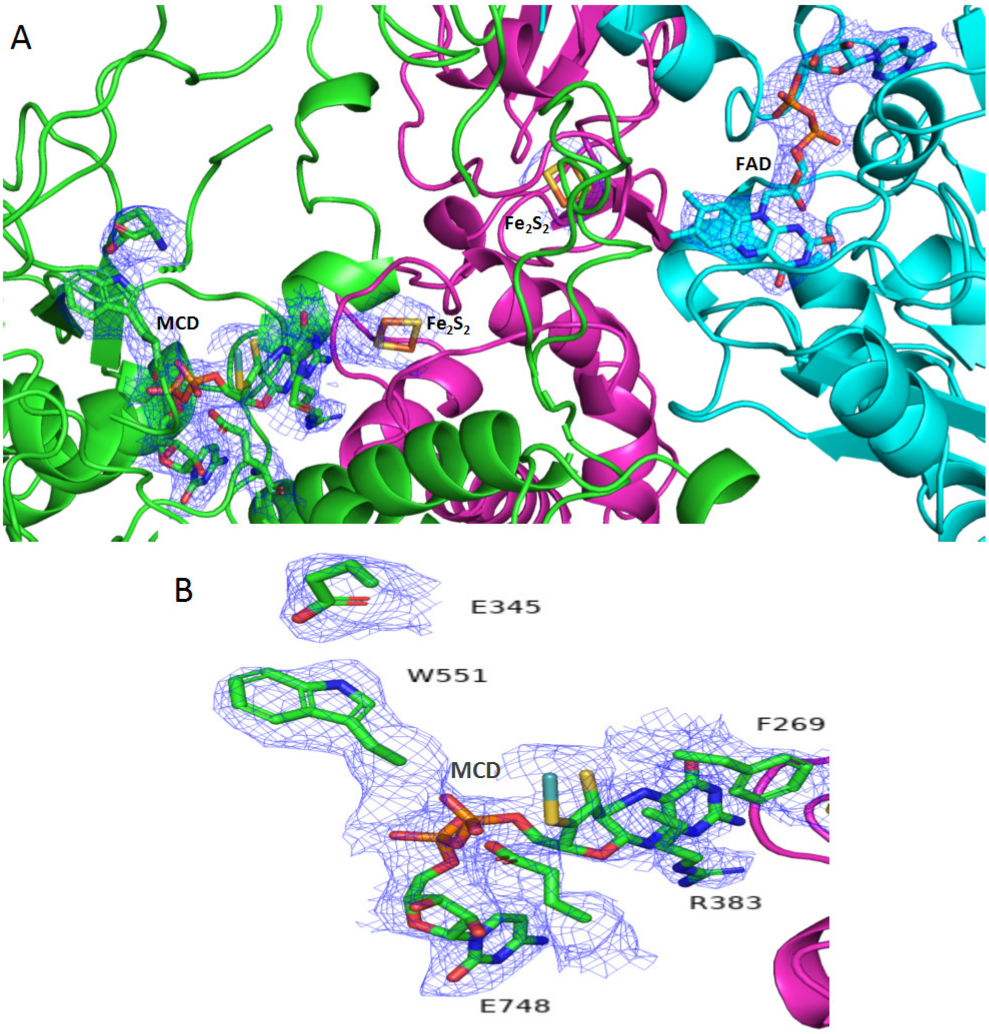

Supplementary Figure 3. Electron density around the Kdh cofactors. (A) Local electron density map of three subunits of cofactors MCD, Fe_2S_2, FAD. (B) Electron density of important amino acids near MCD of the large subunit.

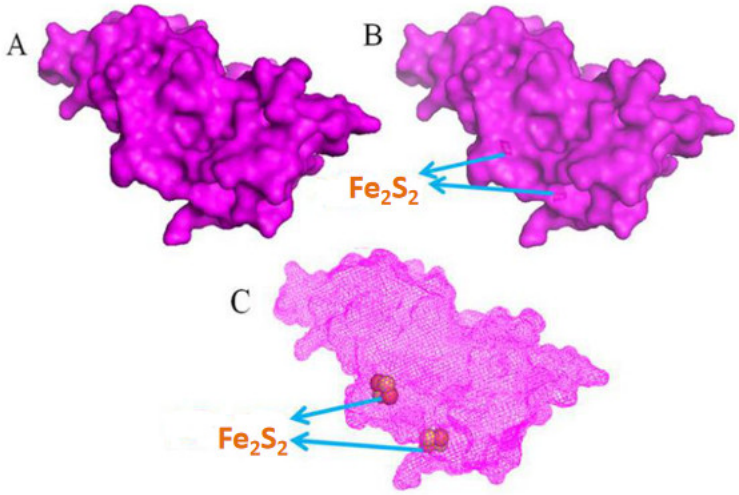

Supplementary Figure 4. The two Fe_2S_2 clusters are completely wrapped inside KdhS (A and B). The Fe_2S_2 clusters cannot be seen when watched from outside the surface of KdhS. KdhS is shown as surface representation while the two Fe_2S_2 clusters are shown as sticks. The Fe_2S_2 clusters cannot be seen if the surface of KdhS is set opaque (A), and can be seen existing inside KdhS if the surface of KdhS is set transparent (B). The Fe_2S_2 clusters are buried inside KdhS (C). KdhS is shown as a mesh presentation, with the Fe_2S_2 clusters inside it.

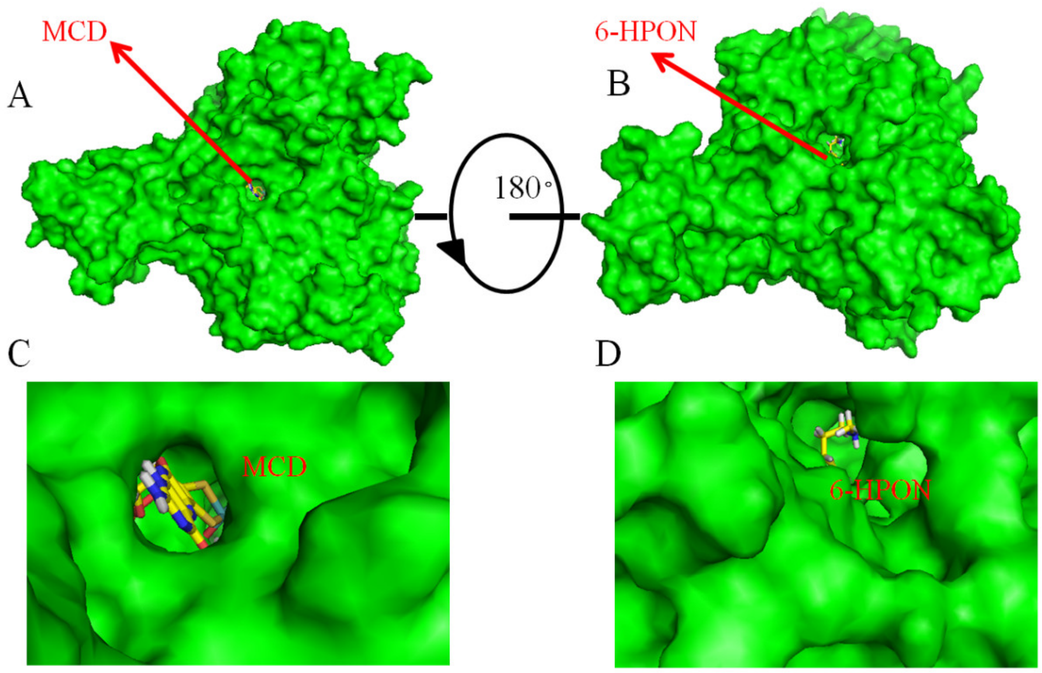

Supplementary Figure 5. KdhL is colored in green and shown as surface representation A and B. Both cofactor MCD and substrate 6-HPON are shown as sticks sitting in the channel, and the MCD can be seen at one end of the channel port (A and C). After turning vertically for 180°, 6-HPON can be seen at the other end of the channel port (B and D). C is a local magnification of A, and D is a local magnification of B.

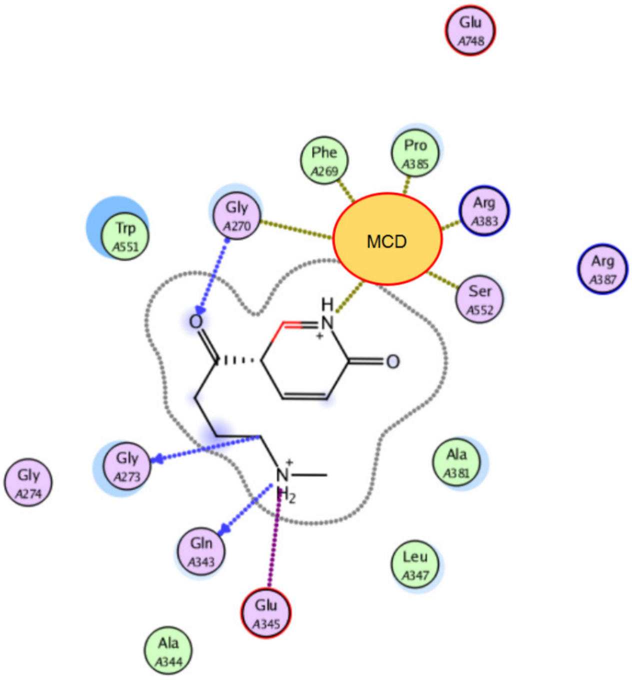

Supplementary Figure 6. Putative the amino acids that interact with MCD and 6-HPON.

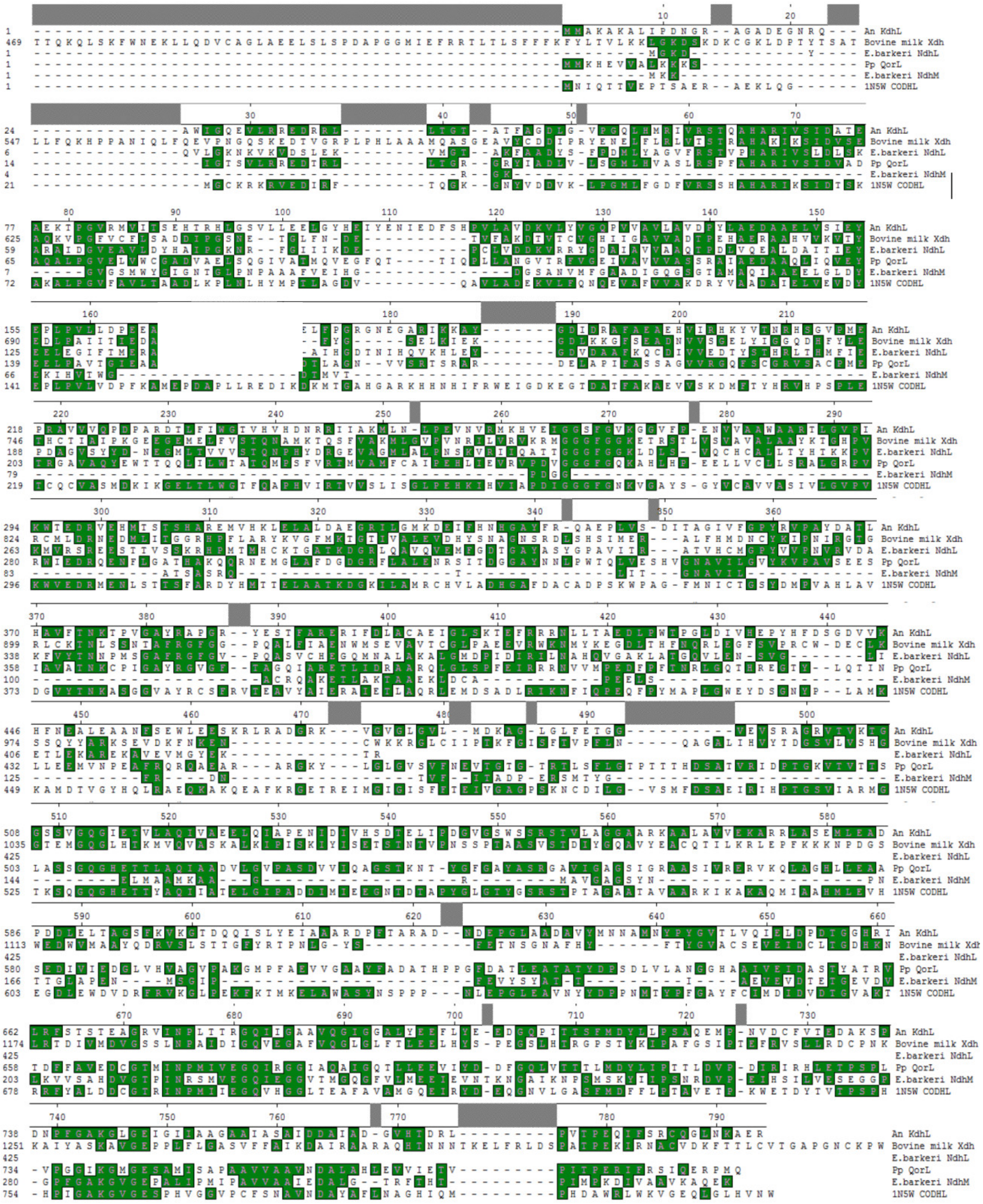

Supplementary Figure 7. Sequence alignment result of different molybdenum hydroxylases. Abbreviations: An KdhL, *Arthrobacter nicotinovorans* KdhL subunit; Xdh: bovine milk Xdh; Pp QorL, *Pseudomonas putida 86* quinoline 2-oxidoreductase

L subunit; Eb NdhL, *Eubacterium barkeri* NdhL subunit; CODH-L, *Oligotropha carboxidovorans* carbon monoxide dehydrogenase L subunit; Eb NdhM, *Eubacterium barkeri* NdhM subunit.

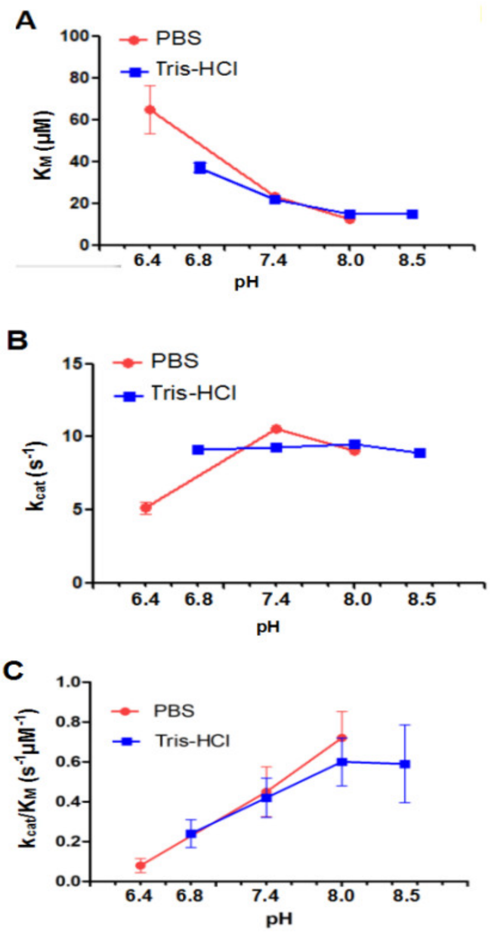

Supplementary Figure 8. The K_M, k_{cat} and k_{cat}/K_M of Kdh in PBS/Tris-HCl buffer of different pH. (A) When the pH of PBS increased from 6.4 to 7.4, the enzyme K_M value increased (red line), when pH 8.0, the kcat decreased. When the pH of Tris-HCl increased from 6.8 to 8.0, the enzyme kcat value increased (B) When the pH of PBS increased from 6.4 to 7.4, the enzyme kcat value increased (red line), when pH 8.0, the kcat decreased. When the pH of Tris-HCl increased from 6.8 to 8.0, the enzyme kcat value increased, however, when Tris-HCl pH 8.5, the kcat decreased a little (blue line). (C) When the pH of PBS increased from 6.4 to 8.0, the enzyme k_{cat}/K_M value increased (red line). When the pH of Tris-HCl increased from 6.8 to 8.0, the enzyme

k_{cat}/K_M value increased, however, when Tris-HCl pH 8.5, the k_{cat}/K_M decreased a little(blue line).

Supplementary Table 1

Strains, plasmids, and primers used in this study

**Strain, plasmid or primer**	**Description or primer sequence**
**Strain**	
Arthrobacter nicotinovorans	The gram-positive soil bacterium
**Plasmids**	
pART2	Kan^{r}, expression vector in pAO1
pART2-*KdhLMS*	Kan^{r}, pART2 containing *KdhLMS*
**Primers**	
KdhL *Bam*HI-F	AAAGGAGTTGGAAATGGATCCATGCATCATCACCATCACCATATGATGGCAAAGGCTAAA
KdhL SalI-R	ACTTGTCCTTGTCAAGACGTCGACTTACCGTTCTGCTTTGTT
KdhMS *Sal*I-F	ACGCGTCGACGTCTTGACAAGGACAAG
KdhMS *Xba*I-R	CTAGTCTAGAGTCGTTGTGATCTCTCTGCAA
pART2-*KdhLMS* -F269A-F	GGCGGCAGTGCGGGGGTGAAGGGCGGAGTCTTCCCAGAA
pART2-*KdhLMS* -F269A-R	CTTCACCCCCGCACTGCCGCCGATCTCTACGTGTTTCAT
pART2-*KdhLMS*	AGGCAGGCGGCGCCGCTAGTCAGCGACATTACTGCCG

-E345A-F	GC
pART2-*KdhLMS*-E345A-R	GACTAGCGGCGCCGCCTGCCTGAAATAGGCACCATGGTT
pART2-*KdhLMS*-R383A-F	GGAGCTTACGCGGCGCCTGGACGCTACGAGTCCACTTTC
pART2-*KdhLMS*-R383A-R	TCCAGGCGCCGCGTAAGCTCCGACAGGTGTTTTGTTGGT
pART2-*KdhLMS*-W551A-F	GTGGGTTCCGCGTCCAGCCGTTCAACTGTTCTTGCTGGAG
pART2-*KdhLMS*-W551A-R	ACGGCTGGACGCGGAACCCACACCGTCCGGAATAAGCTC
pART2-*KdhLMS*-E748A-F	GGCCTTGGGGCGATTGGCATCATTGCGGCCGGCGCGGCA
pART2-*KdhLMS*-E748A-R	GATGCCAATCGCCCCAAGGCCCTTGGCGCCAAAGGGGTT
