# Supplementary figures and images for: Structural, Mechanistic, and Functional Insights into an Arthrobacter nicotinovorans Molybdenum Hydroxylase Involved in Nicotine Degradation
Source: Molecules. 2021 Jul 20;26(14):4387. doi: 10.3390/molecules26144387 (PMC8305194; doi:10.3390/molecules26144387)

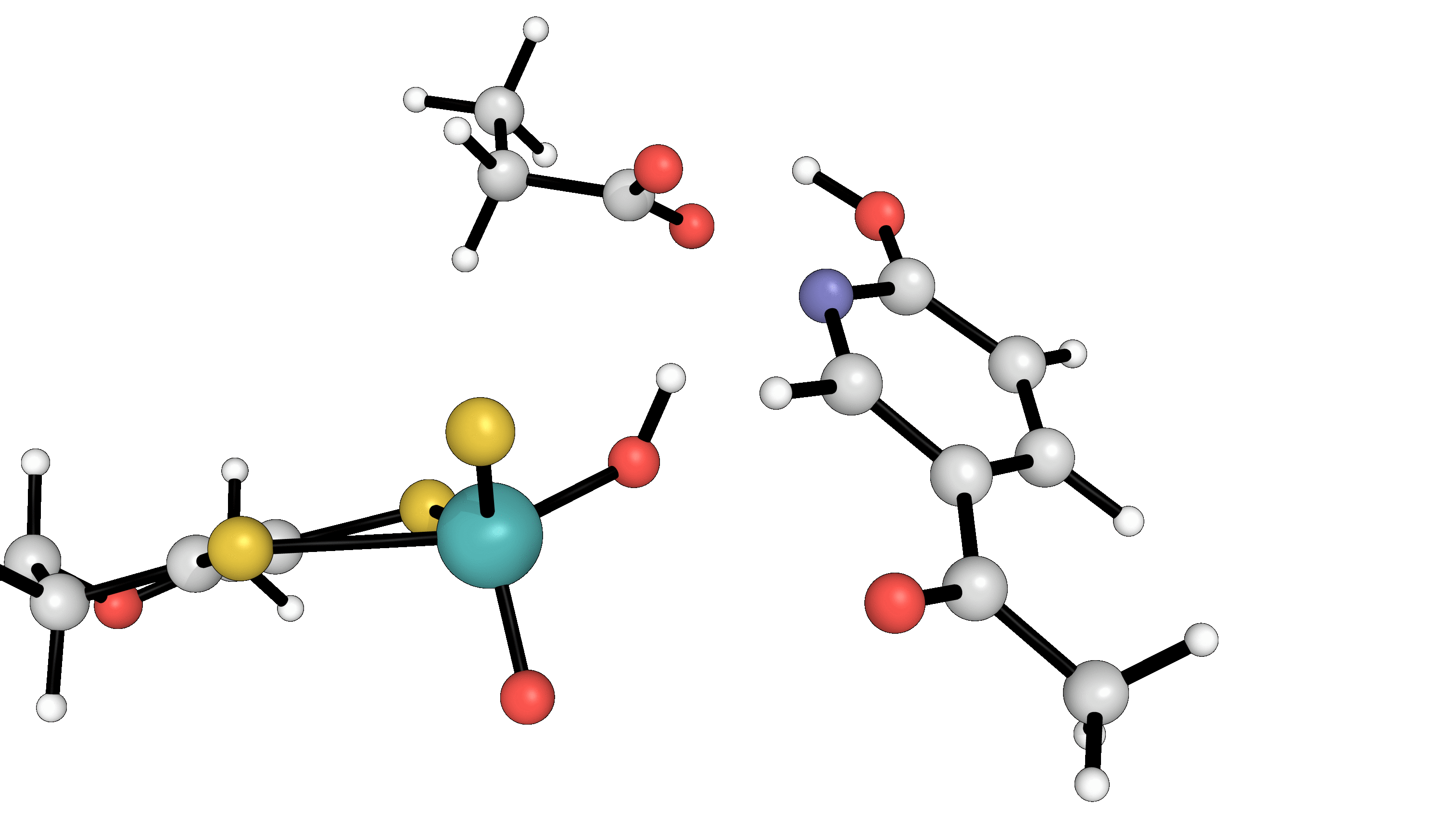

Supplement: Supplementary file 1 [file molecules-26-04387-s001.zip › molecules-1282361 -si/Supplmentary moive.gif]
